# IFN-γ enhances the efficacy of mesenchymal stromal cell-derived exosomes via miR-21 in myocardial infarction rats

**DOI:** 10.1186/s13287-022-02984-z

**Published:** 2022-07-23

**Authors:** Jian Zhang, Yao Lu, Yangming Mao, Yue Yu, Tianyu Wu, Wei Zhao, Yeqian Zhu, Pengcheng Zhao, Fengxiang Zhang

**Affiliations:** 1 Section of Pacing and Electrophysiology, Division of Cardiology, The First Affiliated Hospital of Nanjing Medical University, 300 Guangzhou Road, Nanjing, 210029 People’s Republic of China; 2 Division of Cardiology, Xuzhou Central Hospital, Xuzhou, People’s Republic of China

**Keywords:** MSCs, Exosome, miR-21-5p, IFN-γ, Angiogenesis, Apoptosis

## Abstract

**Background:**

Mesenchymal stromal cells (MSCs) activated with IFN-γ elicit stronger physical effects. Exosomes (Exos) secreted from MSCs show protective effects against myocardial injury. This study aimed to determine whether Exos derived from IFN-γ-treated MSCs exhibit more potent cardioprotective function and the underlying mechanisms.

**Methods:**

H9c2 cells or human umbilical vein endothelial cells (HUVECs) were treated with Exos isolated from MSCs (Ctrl-Exo) or IFN-γ-primed MSCs (IFN-γ-Exo) under oxygen and glucose deprivation (OGD) conditions in vitro and in vivo in an infarcted rat heart. RNA sequencing was used to identify differentially expressed functional transcription factors (TFs). Quantitative reverse transcription-PCR (qPCR) was used to confirm the upregulated TFs and miRNA in IFN-γ-primed MSCs. Dual-luciferase reporter gene assay was used to analyze the transcriptional regulation of miRNAs by STAT1. The target of miR-21-5p (miR-21) was determined by luciferase reporter assays and qPCR. The function of BTG2 was verified in vitro under OGD conditions.

**Result:**

IFN-γ-Exo accelerated migration and tube-like structure formation and prevented OGD-induced apoptosis in H9c2. Similarly, IFN-γ-Exo treatment caused a decrease in fibrosis, reduced cardiomyocyte apoptosis, and improved cardiac function compared to Ctrl-Exo treatment. MiR-21 was significantly upregulated in IFN-γ-primed MSCs and IFN-γ-Exo. STAT1 transcriptionally induced miR-21 expression. Up-regulated miR-21 could inhibit BTG anti-proliferation factor 2 (BTG2) expressions. BTG2 promoted H9c2 cell apoptosis and reversed the protective effects of miR-21 under OGD conditions.

**Conclusion:**

IFN-γ-Exo showed enhanced therapeutic efficacy against acute MI, possibly by promoting angiogenesis and reducing apoptosis by upregulating miR-21, which directly targeted BTG2.

**Supplementary Information:**

The online version contains supplementary material available at 10.1186/s13287-022-02984-z.

## Introduction

IFN-γ, which is produced by activated T cells and natural killer (NK) cells, plays a key role in maintaining innate and adaptive immune responses [1]. It is a cytokine that can promote immunomodulation and has been widely studied for its anti-cancer activity [2]. After interacting with recipient cells through the IFN-γ receptor (IFNGR), IFN-γ activates downstream signal transduction pathways and transcriptionally stimulates the expression of various genes involved in immune regulation and other biological activities [3]. Mesenchymal stromal cells (MSCs) activated with IFN-γ elicit strong immunomodulatory effects via the upregulation of immunoactive factors [4, 5].

MSCs are stromal cells capable of self-renewal and multi-lineage differentiation ability [6] and have been used in regenerative therapy for cardiovascular diseases [7]. The therapeutic effects of MSCs are mainly paracrine and are mediated by Exos [8] Exos are 50–200 nm vesicles secreted into the extracellular space and shuttle a variety of microRNA (miRNA), long non-coding RNA (lncRNA), and proteins to modulate cell–cell communication [9]. MSC-derived Exos have been used to treat ischemic heart disease, inhibit cell apoptosis, promote angiogenesis, and regulate macrophage polarization [10, 11].

Exos derived from modified MSCs exhibit more powerful protective effects. Wang et al. showed that compared with Exos derived from normoxic conditions, Exos derived from hypoxic conditions exerted stronger inhibition of cell death through miR-125b [12]. Adiponectin stimulates the release of Exos to enhance the MSC-mediated treatment of heart failure in mice [13]. Recently, we showed that hypoxia induction and macrophage migration inhibitory (MIF) modification could improve the therapeutic effects of MSC-derived exosomes in myocardial infarction (MI) by inhibiting apoptosis and promoting angiogenesis [14]. IFN-γ treatment can enhance the therapeutic effect of MSCs [15]. However, whether Exos derived from MSCs stimulated by IFN-γ have stronger protective effects against MI than do Exos from untreated MSCs remains unknown.

Ctrl-Exo and IFN-γ-Exo were extracted to treat H9c2 and HUVECs cells under OGD conditions and rat MI models to determine their therapeutic effects and potential mechanisms. We found that IFN-γ treatment could improve the anti-apoptotic ability and angiogenesis of Exos derived from MSCs to preserve heart function. This was partly mediated by the increased expression of cardioprotective miRNA-21 after IFN-γ treatment. Exo-mediated delivery of miRNAs from MSCs under various treatment conditions could prove an effective alternative for MI treatment.

## Materials and methods

### Cell culture

Human umbilical MSCs (purchased from the Clinical Center of Reproductive Medicine of Nanjing Medical University) were cultured in α-minimal essential medium (α-MEM) containing 10% fetal bovine serum (FBS). MSCs were exposed to control or 50 ng/ml IFN-γ (300-02, Peprotech, USA) conditions for 48 h. H9c2 and HUVECswere cultured in Dulbecco's Modified Eagle's Medium (DMEM) containing 10% FBS. All media and reagents used for cell culture were purchased from Gibco (8121231, Carlsbad, USA). For normal culture, cells were incubated at 37 °C, 21% O_2_, and 5% CO_2_. Under oxygen and glucose deprivation conditions, cells were cultured under 1% O_2_, 5% CO_2_, 94% N_2,_ and serum deprivation conditions.

### Exosome extraction and characterization

MSCs (1 × 10^6^) were cultured to 70% confluence and treated with 6 ml of exosome-free FBS (10099141C, Gibco, USA) for 2 d. The cell culture medium (6 ml) was collected in a 15-ml centrifuge tube and centrifuged at 1500×*g* for 30 min to remove the cells and debris. The processed supernatant was transferred to another 15 ml centrifuge tube containing 2 ml RiboTM exosome extraction reagent (for cell culture medium, C10130-2, Ribobio, China). These mixtures were incubated overnight at 4 °C and centrifuged at 2000×*g* for 30 min. The supernatant was discarded, and the exosomal pellet was resuspended in 100 μl PBS.

Exosomal surface markers were detected using western blotting with anti-TSG101, CD63, and CD81 antibodies (14497, 25682, 66866, Abcam, UK). Transmission electron microscopy (TEM) was used to observe the appearance of Exos. The Exos were fixed with 1% glutaraldehyde and coated with 1% phosphotungstic acid on a copper mesh. JEM-2100 transmission electron microscope (JEOL, Tokyo, Japan) was used to observe the sample. Nanoparticle tracking analysis (NTA) was applied to analyze the size and distribution of Exos. We recorded and tracked the Brownian motion of Exos in PBS (Carlsbad, California). The particle size distribution data were obtained using a Stoke-U.S. A ZetaView PMX 110 system (Particle Metrix, Germany) was used for NTA.

### Exosomes uptake assay

To demonstrate the uptake of Exos by H9c2 cells and HUVEC, Exos were labeled with DiI (red fluorescent dye, C1036, Beyotime, China) and co-cultured with recipient cells at 37 °C for 6 or 24 h, washed with PBS, and fixed with 4% paraformaldehyde for 20 min. The nuclei were stained with 6-diamino-2-phenylindole (DAPI) (0.5 g/ml, C1005, Beyotime, China) for 10 min, and observed using a confocal microscope.

### Apoptosis assays

H9c2 cells were seeded in 1 × 10^5^/6-well tissue culture plates overnight and treated with the different Exos or PBS before OGD. The cells were washed with PBS and stained with Annexin V fluorescein isothiocyanate and propidium iodide apoptosis kit (40302ES20, YEASEN, China). Flowjo Software version 10.0 (Tree Star, USA) was used to analyze apoptotic cells. TdT-mediated dUTP Gap End Labeling (TUNEL) Apoptosis Detection Kit (Roche, USA) was also used to detect cell or tissue apoptosis according to the manufacturer's instructions. The formula for calculating the percentage of apoptotic nuclei was the total number of nuclei divided by the total number of TUNEL-positive nuclei.

### Migration assay

HUVECs were cultured in a 6-well plate, and the confluent layer was scraped with the tip of a P200 pipette. The cells were washed and incubated with 100 µg/well of different Exos. Images were taken before and 6 and 12 h after incubation, and Image J software (NIH) was used to determine the reduction ratio of the scratch area.

### Tube formation assay

HUVECs were treated with PBS or different Exos, washed with PBS, and seeded (30,000 cells/well) in 96-well plates coated with growth factor reduced Matrigel (354234, Corning, United States). After 6 h, capillary-like tube formation was observed and photographed. Tube length and number of branches were analyzed with Image J software (NIH).

### Quantitative real-time PCR (qRT-PCR)

The total cellular and exosomal RNA was extracted using Trizol reagent (Life Technologies, USA) according to the manufacturer’s instructions [14]. A stem-loop-specific primer method was used to measure miR-21-5p expression, as described previously [16]. The sequences of primers used in the study are shown in Additional file 2: Table S1. The relative expression was calculated using the following equation: relative gene expression = 2^−(ΔCtsample^ ^−^ ^ΔCtcontrol)^. All samples were measured in triplicate.

### Transfection experiment

Transfection of miR-21-5p mimics (50 nmol/L) and negative control miRNA (50–100 nmol/L) synthesized by Guangzhou Ribobio into H9c2 cells was carried out using riboFECT™ CP Reagent (C11062-1, Ribobio, China) according to the manufacturer’s instructions. The full-length BTG2 sequence and empty vector as negative control were inserted into a pcDNA 3.1 plasmid (GenePharma, China) to transfect H9c2 cells using Lipofectamine 2000 (Invitrogen, USA) according to the manufacturer’s instructions. STAT1 siRNA (Additional file 2: Table S2) and negative control FAM manufactured by Suzhou GenePharma were delivered into MSCs by using Lipofectamine 2000. qRT-PCR was performed to determine transfection efficacy. At 48 h after transfection, different groups of cells were harvested for subsequent experiments.

### Western blot

Protein extraction and western blot (WB) analysis were performed as previously described [14]. Briefly, cells were washed with PBS and lysed with lysis buffer on ice for 20 min. The total cell protein concentration was detected using the BCA Protein Assay Kit. The total protein (20 μg) was separated using SDS-PAGE (Invitrogen) and transferred to a PVDF membrane (Roche). The membrane was blocked with 5% bovine serum albumin (0.1%) in TBS-Tween and incubated against the required antibody.

The primary antibodies Bax (5023, Cell Signaling Technology, USA), Bcl2 (ab196495, Abcam, USA), cleaved caspase-3 (29034, Signalway Antibody, USA), BTG2 (A9848, ABclonal), GAPDH (5174, Cell Signaling Technology), TSG101 (14497, Proteintech, United States), CD63 (25682, Proteintech), CD81 (66866, Proteintech), and horseradish peroxidase-conjugated secondary antibody (Santa Cruz) were used. Bands were visualized using enhanced chemiluminescence reagents and analyzed using a gel documentation system (Bio-Rad Gel Doc1000 and Multi-Analyst version 1.1).

### Ethics statements

Our animal study protocol conforms to the Guide for the Care and Use of Laboratory Animals [National Institutes of Health, (NIH) Bethesda, MD, USA] and was approved by the Institutional Animal Care and Use Committee of the Nanjing Medical University for Laboratory Animal Medicine (IACUC-2005043).

### MI model, histological analysis, and immunofluorescence staining

Eight-week-old male Sprague–Dawley (SD) rats obtained from the experimental animal center of Nanjing Medical University were randomly divided into 4 groups: sham operation group (Sham group, *n* = 6), PBS injection group (MI+PBS group, *n* = 6), Ctrl-Exo injection group (MI+Ctrl-Exo group, *n* = 6), and IFN-γ-Exo injection group (MI+IFN-γ-Exo group, *n* = 6). As previously described [14], the left anterior descending artery (LAD) was ligated, and Exos (50 µL, 1 µg/µL) or PBS was injected around the infarct area in rats. All surgeries and subsequent analyses were blinded for intervention. Echocardiography (Vevo 3100) was performed to determine the left ventricular ejection fraction (LVEF) and left ventricular short-axis shortening rate (LVFS) after 2 w and 4 w.

The rats were sacrificed by cervical dislocation. Inflammatory cell infiltration were evaluated by CD68 immunofluorescence staining at 3 days post-MI. Apoptosis was detected by TUNEL staining (Roche, USA) at 3 days post-MI. Masson's trichrome staining was used to evaluate fibrosis and collagen area, CD31 immunofluorescence staining was applied to observe the distribution of microvessels 4 weeks after MI. The primary antibodies used were anti-CD31 (ab7388; British Abcam) and anti-CD68 (ab283654; British Abcam). DAPI was used for nuclear counterstaining. The images were further analyzed using a fluorescence microscope (Zeiss, Germany) and Image J software (NIH).

### Statistical analysis

Continuous variables and categorical variables were described as mean ± SEM and percentages, respectively. Independent-Sample T-test was used to compare continuous variables between the two groups. One-way Analysis of variance (ANOVA) was used for comparison of three or more groups. All statistical tests were performed using GraphPad Prism software version 8.0, and *P* < 0.05 was considered statistically significant.

## Results

### Characterization of control and IFN-γ-primed MSC-derived Exos

**Fig. 1:**
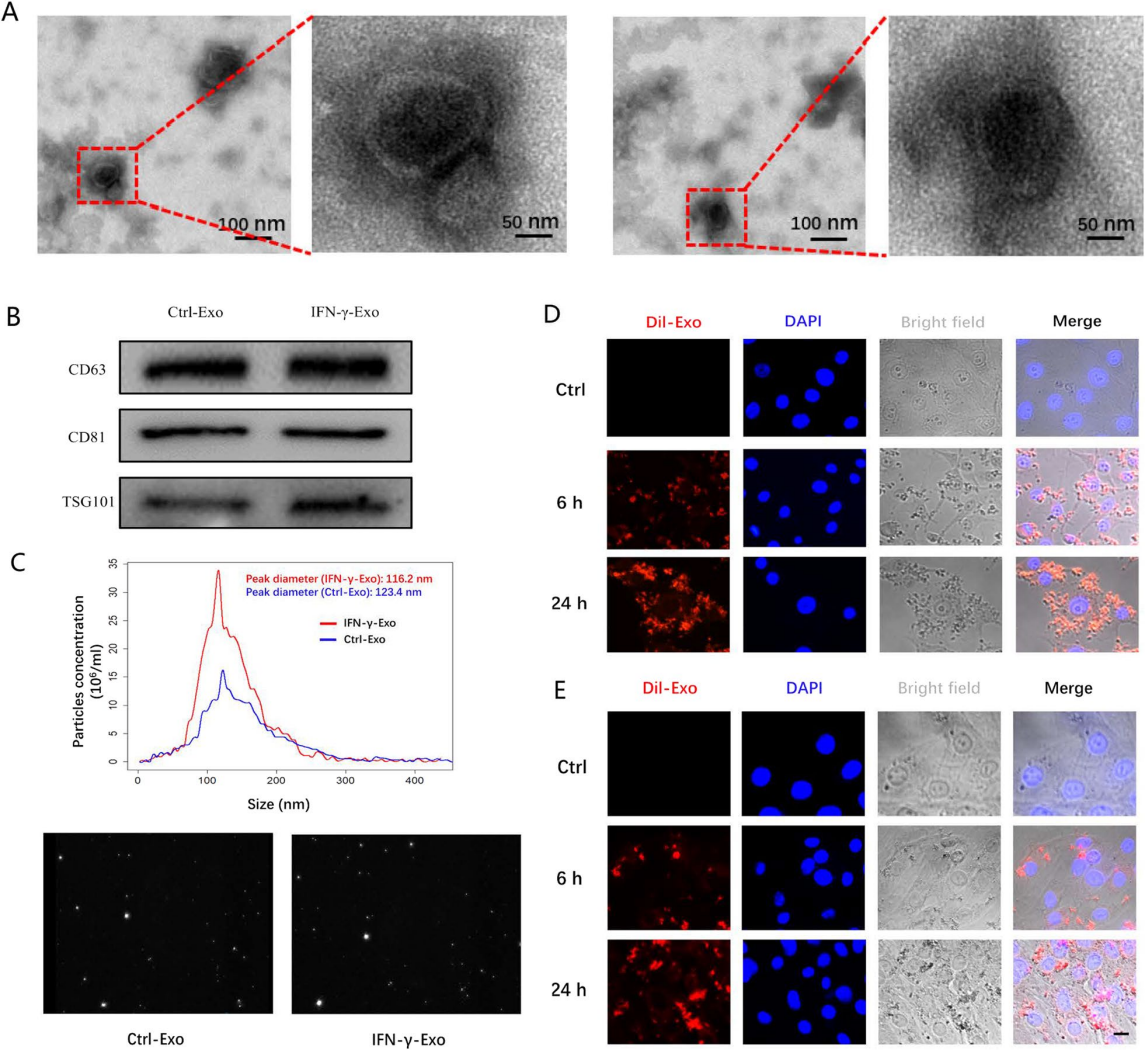
Characterization of Exos derived from MSCs. **A** Cup-shaped morphology of purified Ctrl-Exo and IFN-γ-Exo assessed by TEM. **B** Representative images of western blot showing the exosomal protein markers in Ctrl-Exo and IFN-γ-Exo groups. **C** The particle size distribution and particle concentration were analyzed by nanoparticle tracking analysis (*n* = 3). Confocal images showed that red fluorescence of dye DiI labeled Exos were endocytosed by HUVECs **D** and H9c2 **E** 6 and 24 h after incubation. Scale bar = 20 μm

Exosomes were isolated from supernatant from control MSCs and 50 ng/ml IFN-γ-primed MSCs and identified using TEM. Ctrl-Exo and IFN-γ-Exo were typical lipid bilayer membrane encapsulated nanoparticles with a diameter between 30 and 150 nm (Fig. 1A). The marker proteins TSG101, CD81, and CD63 were expressed in both groups (Fig. 1B). As shown in Fig. 1C, the peak diameters of Exos were 123.4 nm and 116.2 nm in the Ctrl-Exo and IFN-γ-Exo groups, respectively. NTA analysis showed that IFN-γ treatment could increase the number of Exos secreted by MSCs. Similarly, IFN-γ increased the protein concentration in the Exo suspension.

The Exos labeled with DiI dye were co-cultured with HUVEC and H9c2 cells for 6 h and 24 h respectively. Confocal microscopy showed DiI-labeled Exos around the nucleus within 6 h, and most of the Exos were absorbed within 24 h (Fig. 1D, E). Thus, both control MSCs and IFN-γ-primed MSCs could secrete Exos with common vesicle characteristics, and these Exos could be absorbed by H9c2 cells and HUVEC in a time-dependent manner in vitro.

### Pro-angiogenesis and anti-apoptotic effects of IFN-γ-Exo in vitro

**Fig. 2:**
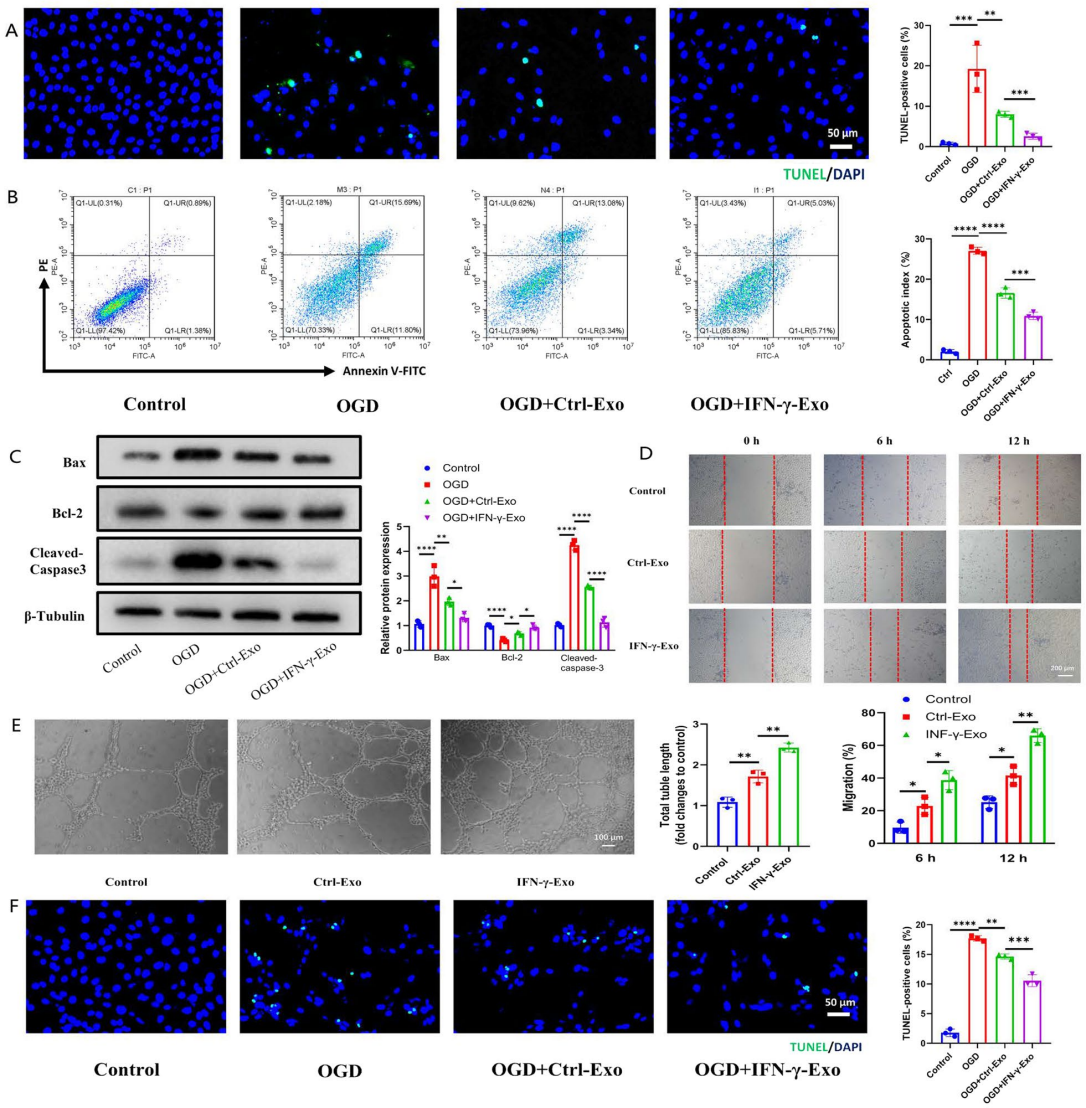
IFN-γ-Exo exhibited better protective effects on HUVECs and H9c2 cardiomyocytes than Ctrl-Exo in vitro. **A** TUNEL analysis for H9c2 cells (*n* = 3). Green, TUNEL-positive nuclei; blue, DAPI-stained nuclei. Scale bars = 50 μm. **B** Flow cytometric analysis (*n* = 3). **C** Western blot analyzed Bax, Bcl2, and cleaved-caspase-3 protein levels in hypoxic and ischemic H9c2 cells incubated with PBS, Ctrl-Exo and IFN-γ-Exo. Relative protein levels are presented as the average expression normalized to β-Tubulin (*n* = 3). **D** Migration was monitored for 6 and 12 h after scratching in HUVECs cultured with PBS, Ctrl-Exo and IFN-γ-Exo (*n* = 3). **E** Tube formation of HUVECs incubated with PBS, Ctrl-Exo, and IFN-γ-Exo (*n* = 3). **F** TUNEL analysis for HUVECs (*n* = 3). Green, TUNEL-positive nuclei; blue, DAPI-stained nuclei. Scale bars = 50 μm. Data are presented as mean ± SEM. Statistical analysis was performed with one-way ANOVA followed by Bonferroni’s correction. *P < 0.05; **P < 0.01; ***P < 0.001, NS not significant

TUNEL staining and flow cytometry showed that the percentage of apoptotic H9c2 cells were significantly lower in the OGD+IFN-γ-Exo group than in the OGD and OGD+Ctrl-Exo groups (Fig. 2A, B). Figure 2C showed that the apoptosis-related proteins Bax and cleaved-caspase-3 were significantly reduced in the OGD+IFN-γ-Exo group, while the anti-apoptotic protein Bcl2 was increased compared with the OGD and OGD+Ctrl-Exo groups. Angiogenesis and migration rate of HUVECs significantly increased in the IFN-γ-Exo group compared with control and Ctrl-Exo groups (Fig. 2D, E). Similar to H9c2 cells, IFN-γ-Exo could better inhibit OGD-induced apoptosis compared with Ctrl-Exo in HUVEC. These results suggested that IFN-γ-Exo confers superior protective effects on H9c2 and HUVECs compared to Ctrl-Exo in vitro.

### IFN-γ-Exo exerted stronger cardioprotection against myocardial damage than Ctrl-Exo in vivo

**Fig. 3:**
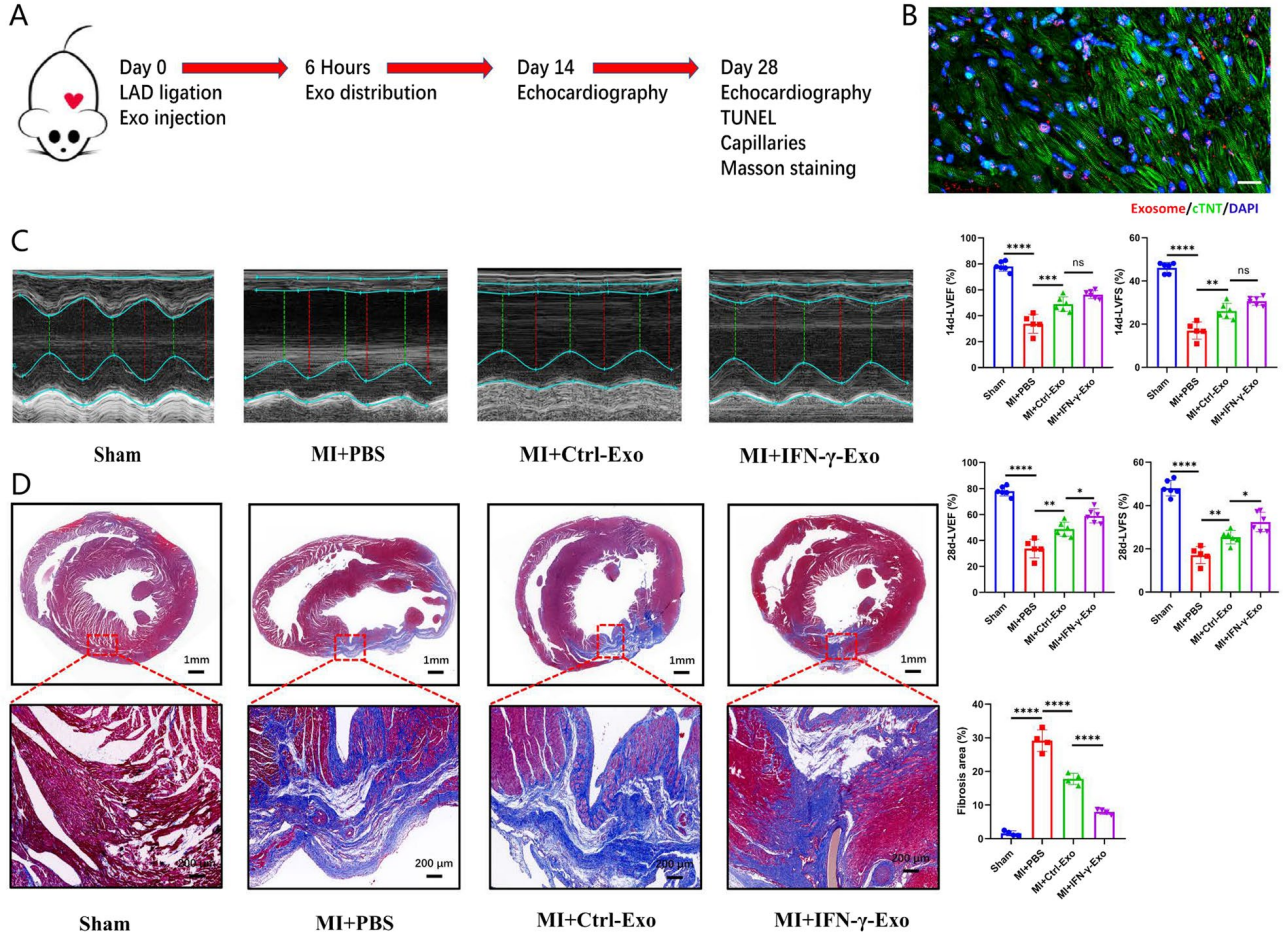
IFN-γ-Exo effectively preserved cardiac function in rats with MI in vivo. **A** The flowchart of experimental design in vivo. **B** DiI-labeled Exos were injected into the infarcted heart of rats for 6 h (50 μg Exos per rat). Representative images of post-MI heart sections stained with DiI-labeled Exos (red), cTNT(green), and DAPI (blue), Scale bar = 20 μm. **C** Representative echocardiographic images showed heart function among the different groups on the 14th and 28th day following MI and quantitative analysis of left ventricular ejection fraction (LVEF) and left ventricular fraction shortening (LVFS) among the different groups (*n* = 6 for the sham group, *n* = 5 for MI+PBS group, *n* = 6 for MI+Ctrl-Exo group, *n* = 6 for MI+IFN-γ-Exo group). **D** Masson’s trichrome stained myocardial sections on the 28th day following MI in rats treated with PBS, Ctrl-Exo, and IFN-γ-Exo. Scar tissue and viable myocardium were identified in blue and red, respectively (*n* = 4). Data were presented as mean ± SEM. Statistical analysis was performed with one-way ANOVA followed by Bonferroni’s correction. *P < 0.05; **P < 0.01; ***P < 0.001; ****P < 0.0001

The AMI rat model was used to determine the cardioprotective effects of MSC-derived Exos. Ctrl-Exo, IFN-γ-Exo (100 µl, 1 µg/µl), or 100 µl PBS were intramyocardially injected at the time of surgery (Fig. 3A). DiI-labeled Exo was intramyocardially injected and colocalized with cardiomyocytes 6 h after injection, suggesting an efficient in vivo uptake of the Exos by heart tissue (Fig. 3B). LVEF and LVFS in 4-weeks’ echocardiography were significantly improved in the MI+IFN-γ-Exo group compared with those in the MI+PBS and MI+Ctrl-Exo groups, while the 2-weeks’ result showed no difference between the MI+IFN-γ-Exo and MI+Ctrl-Exo groups (Fig. 3C). Quantification of the infarct area suggested that IFN-γ-Exo could maximize the reduction of the fibrotic scar area after MI (Fig. 3D). Thus, IFN-γ-Exo has better therapeutic effects against myocardial ischemia and hypoxia injury compared with Ctrl-Exo.

### IFN-γ-Exo inhibits inflammatory cell infiltration and promotes angiogenesis and cardiomyocyte survival in vivo

**Fig. 4:**
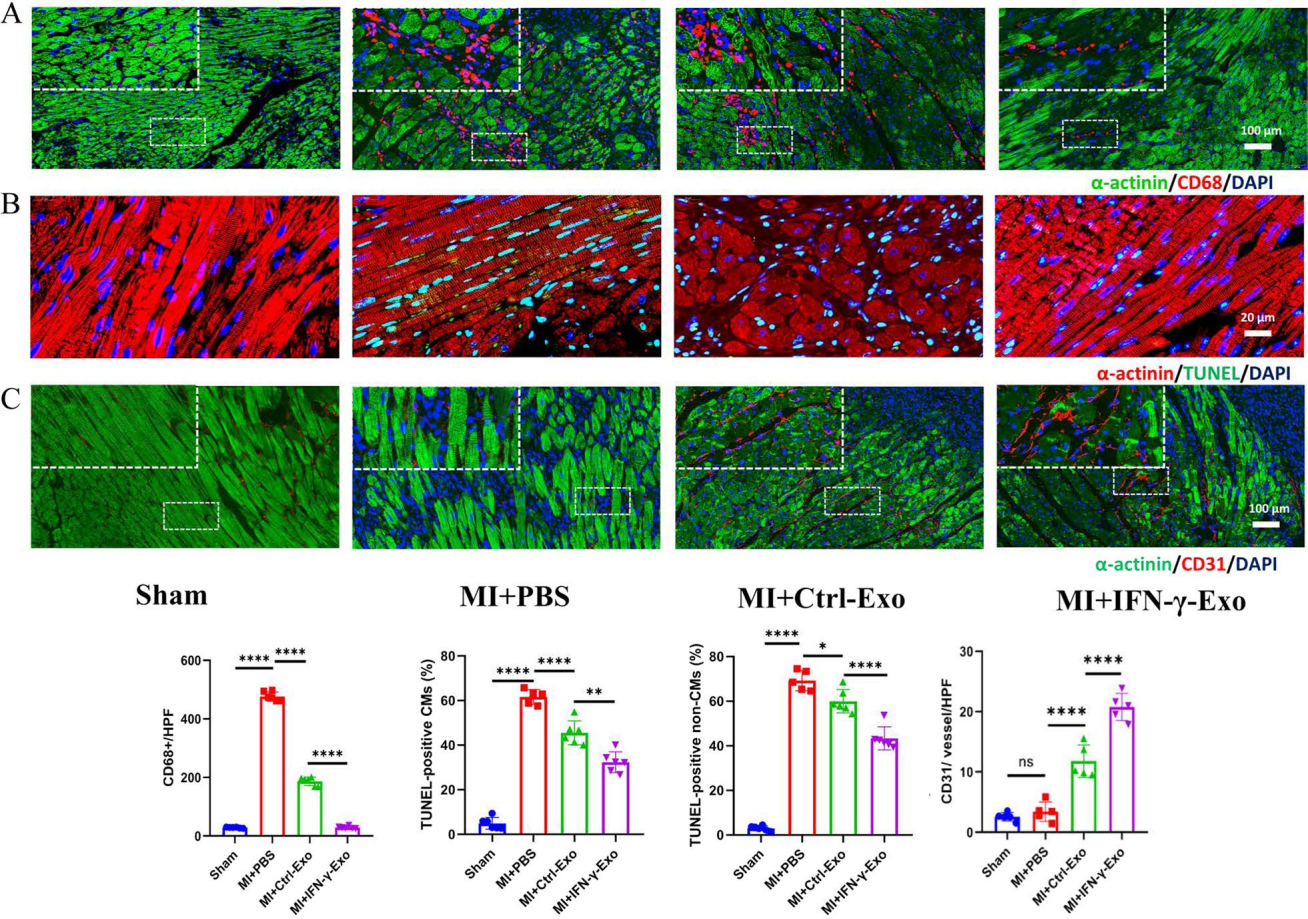
IFN-γ-Exos were better at inhibiting apoptosis or promoting angiogenesis than IFN-γ-Exo in vivo. (**A**) Representative fluorescence images of macrophages in the border zone of ischemic hearts stained with CD68 (red) and α-actinin (green) (3 random fields per anima). (**B**) Representative photographs showing the TUNEL-positive cells (green) in the heart tissue (red) among the different groups. Quantitative analysis of the apoptotic rate at the border zone in CMs and non-CMs among the different groups (3 random fields per animal). (**C**) Representative fluorescence images of blood vessels in the border zone of ischemic hearts stained with CD31 (red) and α-actinin (green) (3 random fields per animal). Data are presented as mean ± SEM. Statistical analysis was performed with one-way ANOVA followed by Bonferroni’s correction. *P < 0.05; **P < 0.01; ***P < 0.001; ****P < 0.0001

CD68 staining showed that the degree of inflammatory cell infiltration in the MI+IFN-γ-Exo group was lower than that of the MI+Ctrl-Exo and MI+PBS groups (Fig. 4A). TUNEL analysis showed that the proportion of apoptotic cells significantly decreased in the MI+IFN-γ-Exo group compared with the MI+PBS and MI+Ctrl-Exo groups (Fig. 4B). CD31 staining showed that the number of regenerative blood vessels in the MI+IFN-γ-Exo group was significantly higher than that in the other two groups (Fig. 4C). Thus, we found that IFN-γ-Exo could exert better protective effects on the myocardium after ischemia and hypoxia injury.

### IFN-γ-Exo attenuates OGD-induced injury in H9c2 cells by upregulating miR‑21 expression

**Fig. 5:**
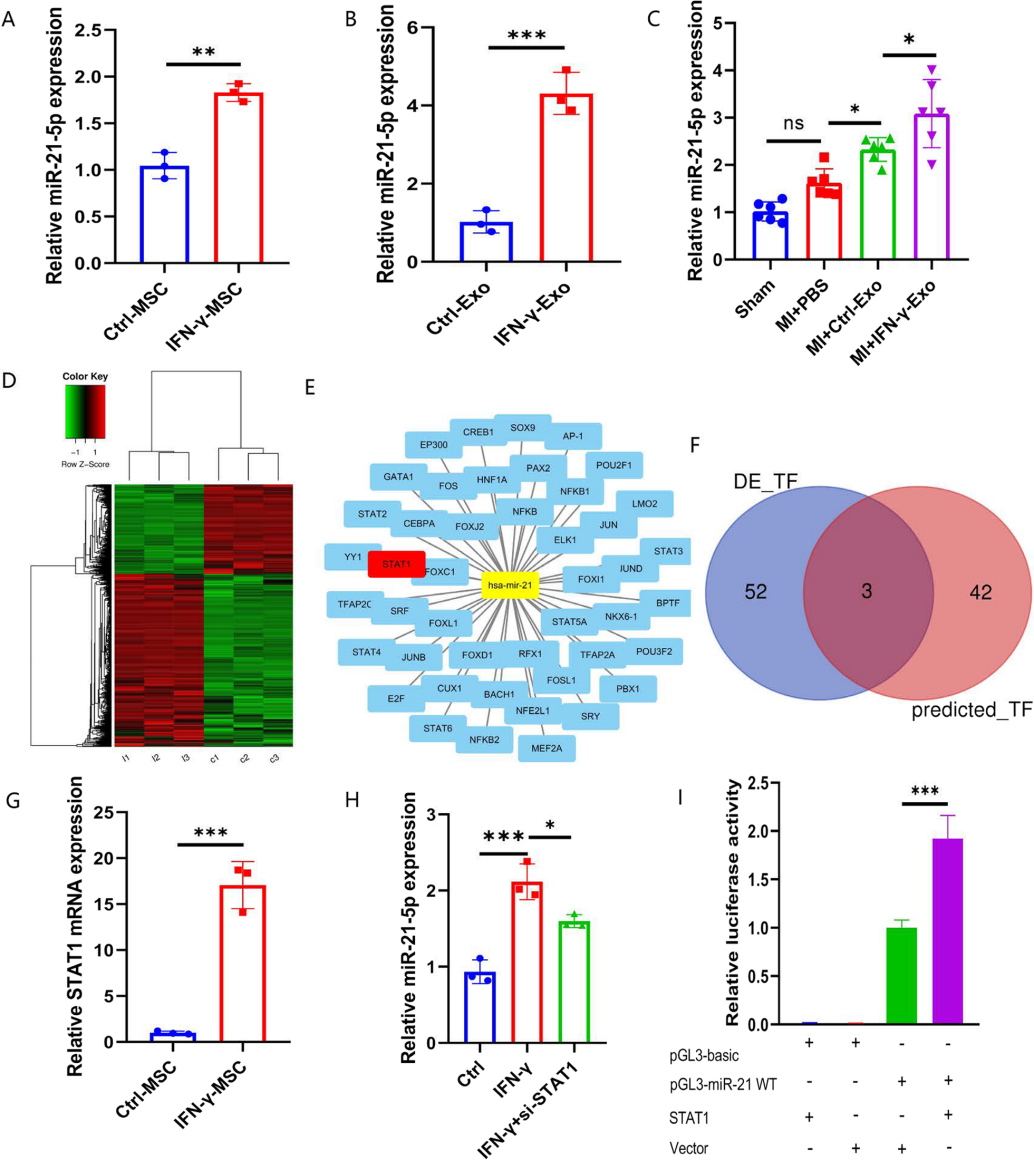
miR-21 was a key component in IFN-γ-Exo-induced cardioprotection*.* Quantitative real-time PCR (qRT-PCR) analysis of miR-21 level in control and IFN-γ-primed MSCs (**A**) and Exos (**B**) (*n* = 3). **C** Quantitative real-time PCR (qRT-PCR) analysis of miR-21 level among groups at 3 days post-MI. (*n* = 6) (**D**) Heat map based on mRNAs sequence values (red represents high expression and the green represents low expression) between control and IFN-γ-primed MSCs. **E** Bioinformatic analysis network of the transcription factors interacting with the promoter of miR‐21. The yellow circle represents hsa‐miR‐21, and the blue and red circles represent transcription factors. **F** STAT1, STAT2, and FOXC1 were present in both up-regulated transcription factors and bioinformatics prediction analysis results. **G** qRT-PCR analysis of STAT1 level in MSCs treated with control and IFN-γ (*n* = 3). **H** qRT-PCR analysis of miR-21-5p level in MSCs treated with control, IFN-γ, IFN-γ + si-STAT1 (*n* = 3). **I** Luciferase reporter assay was used to detect the miR-21 promoter-reporter activity in 293T cells transfected with vector or STAT1 (*n* = 3). Data were presented as mean ± SEM. Statistical analysis was performed with one-way ANOVA followed by Bonferroni’s correction. *P < 0.05; **P < 0.01; ***P < 0.001; ****P < 0.0001

We compared the expression of five putative miRNA in control and IFN-γ-primed MSCs and found (Additional file 1: Fig. S1A) that miR-21 was the most significantly elevated miRNA in IFN-γ-primed MSCs compared with the control ones (Fig. 5A). Further, we found that miR-21 was significantly enriched in IFN-γ-Exo compared with Ctrl-Exo (Fig. 5B). Similarly, the expression level of miR-21 in border myocardial tissue from IFN-γ-Exo group was higher than Ctrl-Exo group (Fig. 5C). To determine the role of miR-21 in regulating apoptosis, miR‑21 mimics and negative control (NC) were transfected into H9c2 cells before exposure to OGD. Moreover, western blot analysis confirmed that the protective effect of miR-21 against OGD-induced injury relied on the upregulation of Bcl2 and downregulation of Bax and cleaved-caspase-3 (Additional file 1: Fig. S1B).

### STAT1 activator promoted miR-21 expression in IFN-γ-primed MSCs

To determine whether transcription factors (TF) were involved in the transcriptional regulation of miR-21, we used Illumina HiSeq 2500 high-throughput sequencing for mRNA expression profiling of control and IFN-γ-primed MSCs to identify the functional TFs in IFN-γ-primed MSCs. With a two fold change and *P* < 0.05 as the threshold cutoff, we identified 55 significantly differentially expressed TFs, of which 37 were upregulated in IFN-γ-primed MSCs (Fig. 5D). Next, we used the TransmiR v2.0 database [17] (http://www.cuilab.cn/transmir) to predict the TFs that might regulate miR-21 (Fig. 5E) and then crossed with the upregulated expressed TFs to finally get 3 potential ones (STAT1, STAT2, and FOXC1) (Fig. 5F). Of these, consistent with sequencing results, STAT1 was the most significantly increased in IFN-γ-primed MSCs compared with control MSCs verified by qPCR (Fig. 5G).

Consistent with previous reports [18, 19], we found that STAT1 was induced by IFN-γ in MSCs and cellular miR-21 was significantly upregulated. Further, the IFN-γ induction of miR-21 was abolished following STAT1 downregulation (Fig. 5H). In addition, we cloned the wild-type fragment miR-21 promoter into the upstream region of the luciferase reporter gene (pGL3-basic) to obtain pGL3-miR-21 WT and found that pGL3-miR-21 WT was activated by STAT1 overexpression. These results suggested potential transcriptional regulation of miR-21 by STAT1 (Fig. 5I).

### BTG2 is a target gene of miR-21 and promotes H9c2 cell apoptosis under OGD

**Fig. 6:**
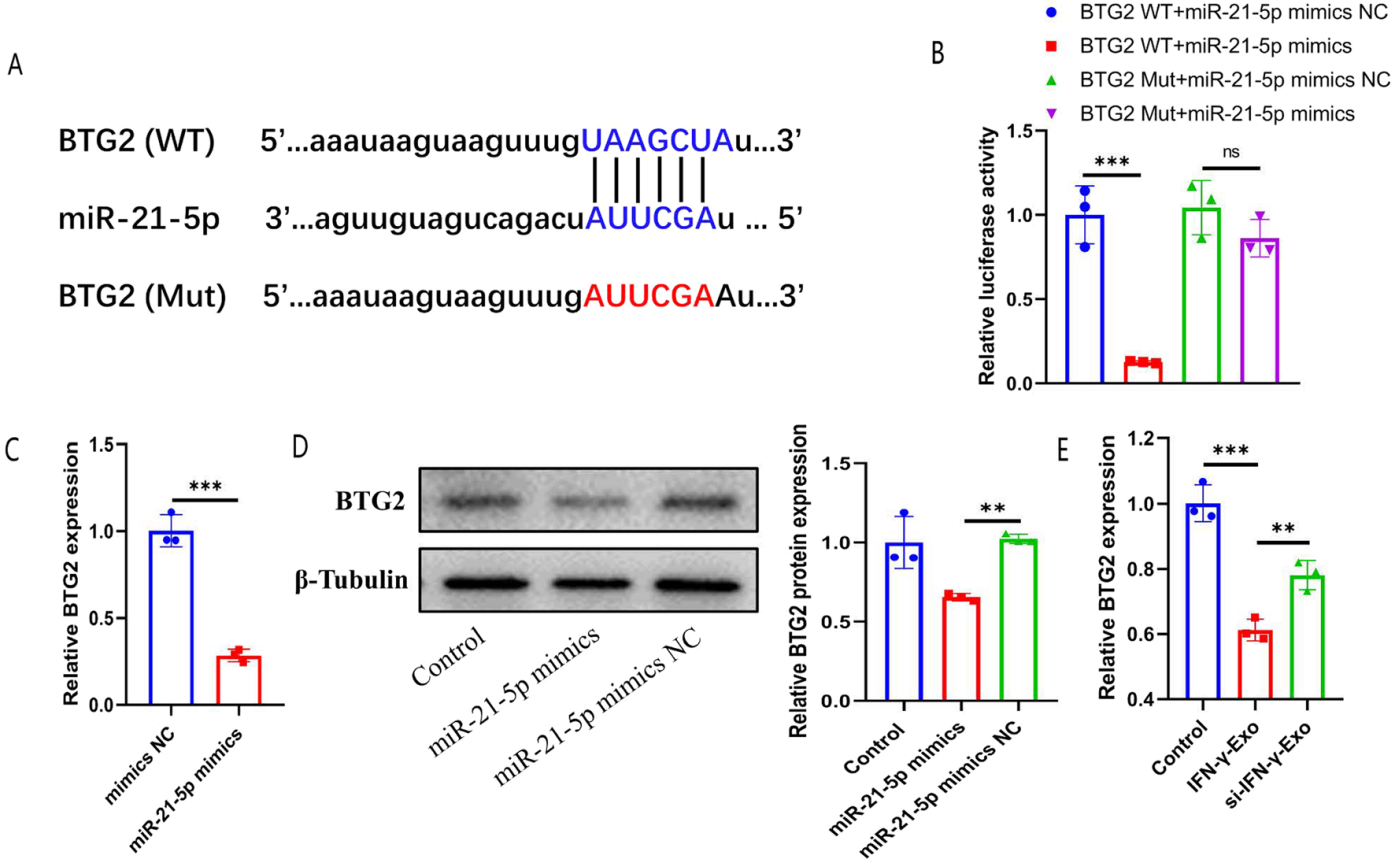
MiR-21 directly targeted BTG2 in H9c2 cells. **A** Predicted miR-21 target sequence in BTG2-3′ UTRs. Target sequences of BTG2-3′ UTRs were mutated. **B** Luciferase assay of 293T cells transfected with BTG2-3′ UTR-WT or BTG2-3′ UTR-Mut reporter together with mimics NC or miR-21-5p mimics (*n* = 3). **C** qRT-PCR analysis of BTG2 level in H9C2 cells treated with mimics NC and miR-21-5p mimics (*n* = 3). **D** Western blot analyzed BTG2 protein levels in H9c2 cells treated with control, mimics NC and miR-21-5p mimics. Relative protein levels were presented as the average expression normalized to β-Tubulin (*n* = 3). **E** qRT-PCR analysis of BTG2 level in H9c2 cells treated with IFN-γ-Exo and si-IFN-γ-Exo (*n* = 3). Data were presented as mean ± SEM. Statistical analysis was performed with one-way ANOVA followed by Bonferroni’s correction. *P < 0.05; **P < 0.01; ***P < 0.001; ****P < 0.0001

Using TargetScan and miRDB, BTG2 was identified as a potential target for miR‑21 (Fig. 6A). Luciferase reporter assays confirmed the association between miR-21 and BTG2: the luciferase activity of BTG2-wt in miR-21 transfected cells was significantly inhibited, while that of the BTG2-mut remained unchanged (Fig. 6B). Furthermore, the mRNA and protein levels of BTG2 were significantly decreased in the miR-21 mimics groups (Fig. 6C, D). In vivo experiments showed that both IFN-γ-Exo and Ctrl-Exo could down-regulate the expression of BTG2 in myocardial tissue after infarction, and IFN-γ-Exo had a better inhibitory effect (Additional file 1: Fig. S1C–D). After co-incubating the exosomes derived from IFN-γ-primed MSCs (IFN-γ-Exo) and IFN-γ-primed + si-STAT1-MSCs (si-IFN-γ-Exo) with H9c2, qPCR results showed that the expression of BTG2 decreased in the IFN-γ-Exo group, but the decrease in the si-IFN-γ-Exo group was not as obvious as in IFN-γ-Exo group (Fig. 6E). Thus, there is a negative regulatory relationship between miR-21 and BTG2.

**Fig. 7:**
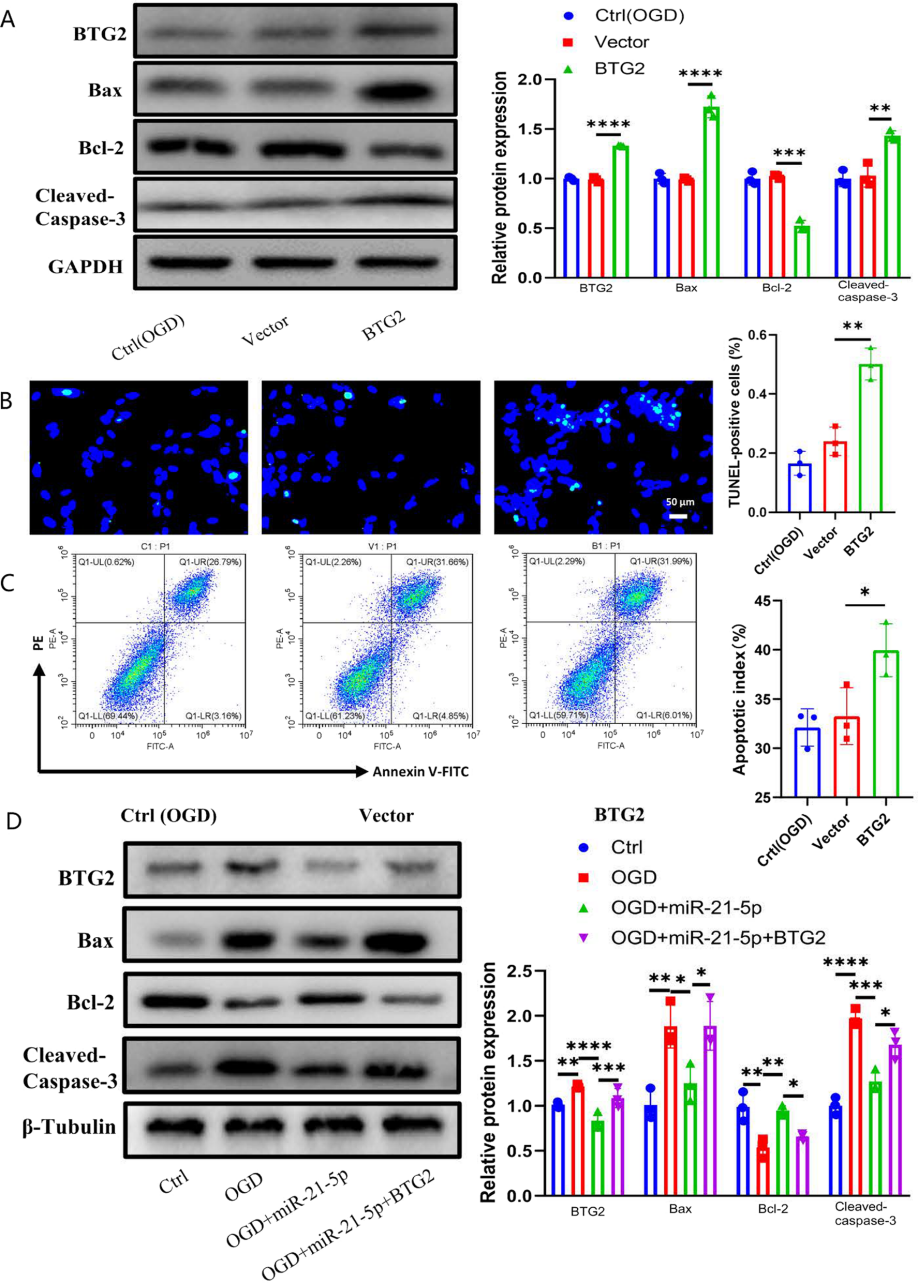
Overexpression of BTG2 aggravated OGD-induced injury damage and reversed the protective effect of miR-21. **A** Western blot analyzed Bax, Bcl2, and cleaved-caspase-3 protein levels in hypoxic and ischemic H9c2 cells treated with PBS, vector and BTG2. Relative protein levels were presented as the average expression normalized to GAPDH (*n* = 3) and TUNEL analysis (*n* = 3) **B** Green, TUNEL-positive nuclei; blue, DAPI-stained nuclei. Scale bars = 50 μm. And flow cytometric analysis (*n* = 3) (**C**). **D** H9c2 cells treated with control, OGD, OGD  + miR-21 mimics and OGD  + miR-21 mimics + BTG2. Relative Bax, Bcl2, and cleaved-caspase-3 protein levels among different groups were presented as the average expression normalized to β-Tubulin (*n* = 3). Data were presented as mean ± SEM. Statistical analysis was performed with one-way ANOVA followed by Bonferroni’s correction. *P < 0.05; **P < 0.01; ***P < 0.001; ****P < 0.0001

Subsequently, BTG2 was overexpressed to determine its effect on H9c2 cell apoptosis under OGD conditions. As indicated in Fig. 7a, western blot analysis showed that BTG2 was successfully overexpressed in H9c2 cells via transfection with BTG2 plasmid. Further experiments revealed that apoptosis induced by OGD was further aggravated by BTG2 overexpression. The expression of Bax and cleaved-caspase-3 protein increased significantly in the BTG2 group, while the expression of Bcl2 protein decreased (Fig. 7A). The same trend was verified by TUNEL staining and flow cytometry; the incidence of apoptosis in the BTG2 group was significantly higher than that in the ctrl (OGD) and vector groups (Fig. 7B, C).

To elucidate the functional relationship between miR-21 and BTG2, we studied the effect of miR-21 mimics and BTG2 plasmid on H9C2 under OGD conditions. Consistent with the previous findings [20], ischemia and hypoxia increase the expression of Bax and cleaved-caspase-3 and also the expression of BTG2, which is inhibited by miR-21 mimics. However, the anti-apoptotic effect of miR-21 on H9C2 cells under OGD conditions was abrogated by BTG2 overexpression (Fig. 7D). These results indicated that the overexpression of BTG2 reverses the protective effect of miR-21 on OGD-treated H9c2 cells.

## Discussion

**Fig. 8:**
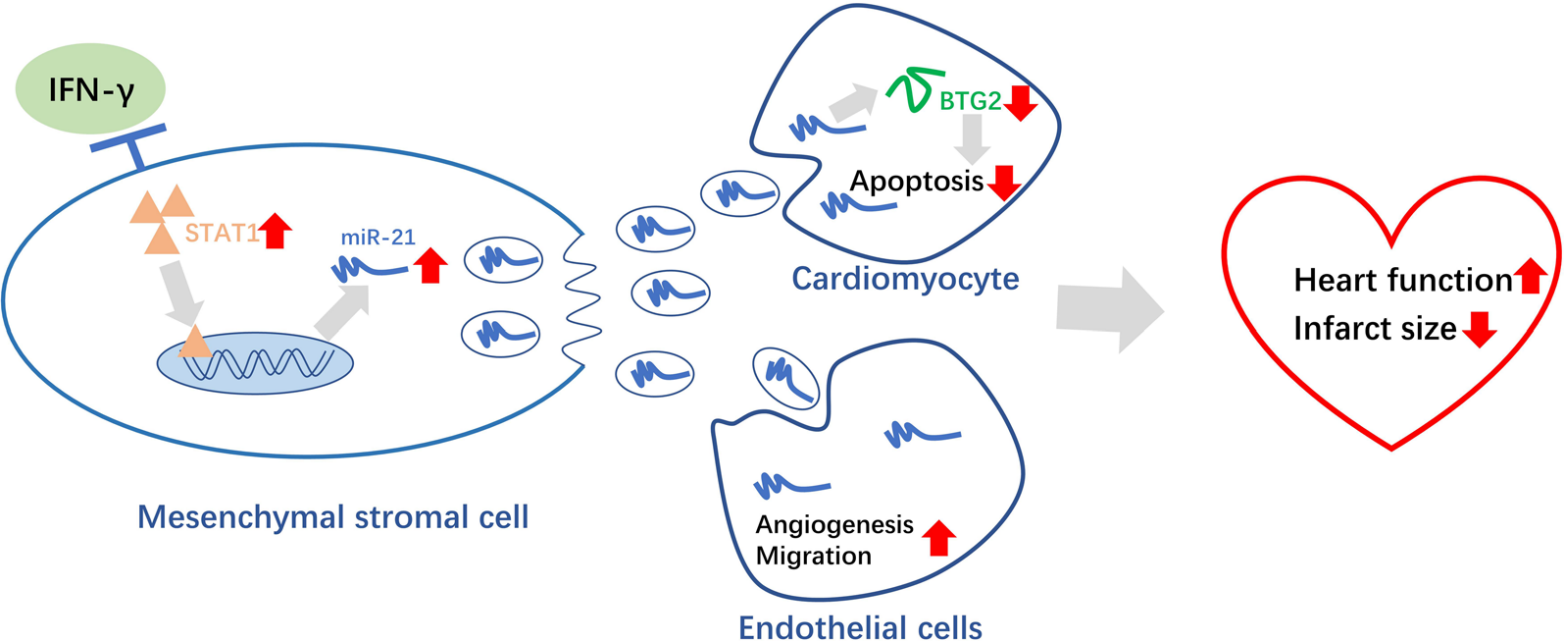
Exosomal miR-21 derived from IFN-γ-primed MSCs improved cardiac function after infarction by promoting angiogenesis and inhibiting apoptosis

In this study, we found that compared with Ctrl-Exo, IFN-γ-Exo accelerated migration and tube-like structure formation, and protected H9c2 from OGD-induced apoptosis. IFN-γ-Exo treatment also reduced fibrosis size and cardiomyocytes apoptosis and improved recovery in cardiac function. Further, IFN-γ-Exo attenuates OGD-induced injury in H9c2 cells by upregulating miR‑21 expression stimulated by STAT1, which directly targets BTG2 (Fig. 8).

There is growing evidence that Exos released from MSCs can protect ischemic cardiomyocytes from death, improve ventricular remodeling, and preserve heart function [21]. The improvement of the therapeutic effects of Exos is therefore of great interest. There have been several studies on modification methods that can reinforce or improve the therapeutic effect of MSC-derived Exos. Hypoxia-treated MSC-derived Exos exhibit more effective cardioprotection mediated by UCA1 and miR-125b [12, 16]. Genetic modification could also enhance the treatment of heart disease by adjusting the expression of various miRNA. Exos secreted by MSCs overexpressing GATA-4 retain a large amount of anti-apoptotic miRNA for cardioprotection [22]. Exos derived from MSCs modified by MIF could be used to treat acute MI by limiting apoptosis and promoting angiogenesis [14]. Some drugs and cytokines that improve the characteristics of stem cells have also been identified. Atorvastatin as a common clinical lipid-lowering drug increases the therapeutic effect by up-regulating the expression of H19 [23]. IFN-γ, an immune-related cytokine, mediates enhanced immunomodulation efficacy in MSC-derived Exos and therefore has better efficacy in the treatment of colitis. Our research showed that Exos derived from IFN-γ-primed MSCs have better anti-apoptosis and angiogenesis effects compared with the control group in cardiomyocytes under OGD conditions and MI rat models. These results indicated that IFN-γ treatment could be used to improve the therapeutic effect of exosomes derived from MSCs.

Minimizing the loss of myocardial cells and restoring the microcirculation in the marginal zone of the infarction are common strategies for the treatment of MI [24]. miR-21 is a typical cardioprotective miRNA, which has therapeutic effects such as anti-apoptosis, anti-fibrosis, and inhibiting inflammation [25]. Subsequently, to explore the mechanism of miR-21 upregulation, we used RNA sequencing to identify the transcription factors that were upregulated after IFN-γ treatment and selected STAT1 as the factor that may regulate the expression of miR-21. STAT3 can stimulate the expression of miR-21 by binding to the promoter region [26, 27]. As an important member of the signal transduction and transcription activator protein family, STAT1 has a similar structure and function as STAT3 [28]. Our results revealed that STAT1 induced by IFN-γ could upregulate miR-21 expression by binding to the promoter region.

The angiogenesis effect of miR-21 has been extensively studied. We further explored the anti-apoptotic effect of miR-21 under ischemia and hypoxia [29, 30]. BTG2 (BTG anti-proliferation factor 2) is the first gene found in the BTG/TOB gene family and exerts a tumor suppressor effect in various cancers [31]. Down-regulation of BTG2 by miR-21 can protect cardiomyocytes from doxorubicin treatment [32]. Functional studies have shown that overexpression of BTG2 can exacerbate H9c2 cell apoptosis under ischemic and hypoxic conditions. Moreover, BTG2 reversed the protective effect of miR-21 on hypoxia-induced injury in H9c2 cells. BTG2 was found to be up-regulated under OGD conditions, which suggests that BTG2 plays an important role in myocardial infarction. Notably, we employed transfection reagents to transiently regulate the expression of target genes. This method cannot achieve long-term effect, so the use of adenovirus transfection will make the results more stable.

Apart from miR-21, MSC-derived exosomes also deliver a wide spectrum of miRNAs that might mediate cardioprotective effects. We found that the expression of miR-126 also increased in IFN-Exo. MiR-126 carried by exosomes promotes angiogenesis and attenuates apoptosis in vivo [33]. In addition, recent studies have shown that miRNAs, proteins and mitochondria encapsulated by exosomes could also exert cardioprotective effects [34]. The therapeutic efficacy of IFN-Exo is likely mediated by a combination of multiple factors, which merit further study.

The present study has some limitations. First and foremost, due to a limited budget, we did not perform miRNA sequence exosomes between Ctrl-Exo and IFN-Exo. It is recommended that gene sequencing can comprehensively reveal the miRNA and lncRNA profile of IFN-Exo. Although miR-21 mainly mediates the cardioprotective effect of IFN-Exo, there are still other miRNA interactions. Therefore, the miRNA sequence of IFN-Exo can better elucidate the cardioprotective efficacy. Second, although intramyocardial injection is an effective method of delivering therapeutic components accurately to the target, it might cause myocardial injury and fluid leakage. Chemical or genetic modification of extracellular vesicles to improve myocardial targeting may be a promising solution.

## Conclusion

In this study, we have shown that Exos derived from control and IFN-γ-primed MSCs attenuate myocardial injury. IFN-γ-Exo showed superior therapeutic efficacy, which was mainly mediated by increased expression of miR-21-5p. Our findings provide insight into the mechanism underlying the anti-apoptotic and angiogenic effects of MSC-derived Exos and suggest a potential strategy to treat ischemic heart disease.

## Supplementary Information

**Additional file 1. Fig. S1**: (A) Expression of putative functional miRNA between control and IFN-γ-primed MSCs. MiR-21 and miR-126 were upregulated in IFN-γ-primed MSCs while miR-424, miR-30b, and miR-30c showed no significant difference between the two groups (n = 3). (B) Western blot analyzed Bax, Bcl2, and cleaved-caspase-3 protein levels in hypoxic and ischemic H9c2 cells. Relative protein levels were presented as the average expression normalized to β-Tubulin (n = 3). Quantitative real-time PCR (qRT-PCR) (C) and western blot (D) analysis of BTG2 level among groups at 3 days post-MI (n = 6). (E) The knockout efficiency of si-STAT1. QRT-PCR analysis of STAT1 level in MSCs treated with negative control and STAT1 siRNA at 24 h after transfection. (F) Western blot analyzed STAT1 protein level in MSCs treated with negative control and STAT1 siRNA at 6、12、24 h after transfection. Data are presented as mean ± SEM. Statistical analysis was performed with one-way ANOVA followed by Bonferroni’s correction. *P < 0.05, **P < 0.01, ***P < 0.001, ****P < 0.0001.

**Additional file 2. Table S1**: Real-time PCR primers. **Table S2**: The sequence of si-STAT1.

## Data Availability

The data that support the finding of this study are available from the corresponding upon reasonable request.

## Abbreviations

MSC: Mesenchymal stromal cells
IFN: Interferon
Exos: Exosomes
CM: Cardiomyocyte
OGD: Oxygen and glucose deprivation
MI: Myocardial infarction
TF: Transcription factors
STAT1: Signal transducer and activator of transcription 1
BTG2: BTG anti-proliferation factor 2
Bcl2: BCL2 apoptosis regulator
Bax: BCL2 associated X, apoptosis regulator
HUVEC: Human umbilical vein endothelial cells
