# Supplementary figures and images for: IFN-γ enhances the efficacy of mesenchymal stromal cell-derived exosomes via miR-21 in myocardial infarction rats
Source: Stem Cell Res Ther. 2022 Jul 23;13:333. doi: 10.1186/s13287-022-02984-z (PMC9308256; doi:10.1186/s13287-022-02984-z)

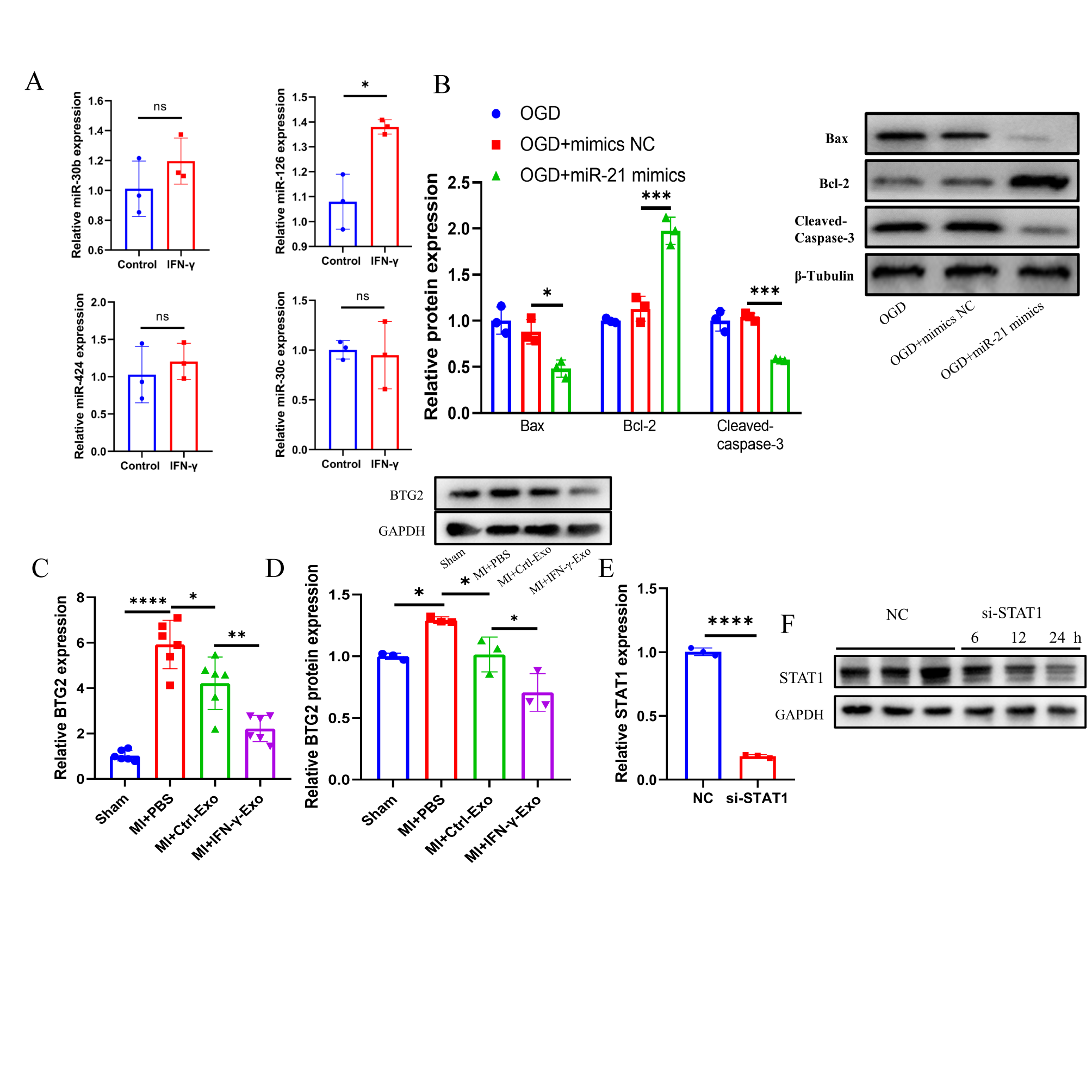

Supplement: Supplementary file 1 — Additional file 1. Fig. S1: (A) Expression of putative functional miRNA between control and IFN-γ-primed MSCs. MiR-21 and miR-126 were upregulated in IFN-γ-primed MSCs while miR-424, miR-30b, and miR-30c showed no significant difference between the two groups (n = 3). (B) Western blot analyzed Bax, Bcl2, and cleaved-caspase-3 protein levels in hypoxic and ischemic H9c2 cells. Relative protein levels were presented as the average expression normalized to β-Tubulin (n = 3). Quantitative real-time PCR (qRT-PCR) (C) and western blot (D) analysis of BTG2 level among groups at 3 days post-MI (n = 6). (E) The knockout efficiency of si-STAT1. QRT-PCR analysis of STAT1 level in MSCs treated with negative control and STAT1 siRNA at 24 h after transfection. (F) Western blot analyzed STAT1 protein level in MSCs treated with negative control and STAT1 siRNA at 6、12、24 h after transfection. Data are presented as mean ± SEM. Statistical analysis was performed with one-way ANOVA followed by Bonferroni’s correction. *P < 0.05, **P < 0.01, ***P < 0.001, ****P < 0.0001. [file 13287_2022_2984_MOESM1_ESM.tif]
