# Supplementary material for: IFN-γ enhances the efficacy of mesenchymal stromal cell-derived exosomes via miR-21 in myocardial infarction rats
Source: Stem Cell Res Ther. 2022 Jul 23;13:333. doi: 10.1186/s13287-022-02984-z (PMC9308256; doi:10.1186/s13287-022-02984-z)
Supplement: Supplementary file 2 — Additional file 2. Table S1: Real-time PCR primers. Table S2: The sequence of si-STAT1. [file 13287_2022_2984_MOESM2_ESM.doc]

**IFN-γ enhances the efficacy of exosomes derived from mesenchymal stem cells in myocardial infarction rats via miR-21 stimulated by STAT1**

Jian Zhang†, Yao Lu†, Yangming Mao†, Yue Yu, Tianyu Wu, Wei Zhao, Yeqian Zhu, Pengcheng Zhao and Fengxiang Zhang*

Section of Pacing and Electrophysiology, Division of Cardiology, the First Affiliated

Hospital with Nanjing Medical University, Nanjing, China;

†The authors contributed equally to this study

**Running title:** IFN-γ enhances the efficacy of exosomes from MSCs

Address for correspondence:

Prof. Fengxiang Zhang,

Section of Pacing and Electrophysiology, Division of Cardiology, the First Affiliated Hospital of Nanjing Medical University, Guangzhou Road 300, Nanjing, 210029, PR

China

Tel. NO./Fax: +86-25-83717168.

E-mail address: [njzfx6@njmu.edu.cn](mailto:njzfx6@njmu.edu.cn)

**Table S1** Real time PCR primers

| Primers | Sequences (5'–3') |
| --- | --- |
| hsa-miR-21-5p Forward | GCGCGTAGCTTATCAGACTGA |
| hsa-miR-21-5p Reverse | AGTGCAGGGTCCGAGGTATT |
| hsa-miR-21-5p  RT  hsa-miR-126-3p Forward  hsa-miR-126-3p Reverse  hsa-miR-126-3p RT  hsa-miR-424-3p  Forward  hsa-miR-424-3p  Reverse  hsa-miR-424-3p  RT  hsa-miR-30b  Forward  hsa-miR-30b  Reverse  hsa-miR-30b  RT  hsa-miR-30c  Forward  hsa-miR-30c  Reverse  has-miR-30c  RT  U6 Forward | GTCGTATCCAGTGCAGGGTCCGAGGTATTCGCACTGGATACGACTCAACA  AGGGTCGTACCGTGAGTAAT  GCTGTTGTGTTGTGTTGTGG  GGCTGTTGTGTTGTGTTGTGGATACAACAGCCCGCATT  GCAAAACGTGAGGCGC  GTGTGGTGTGGTATGGTGTG  GGTGTGGTGTGGTATGGTGTGATCACCACACCATAGCA  AGGGGTGTAAACATCCTACAC  TCCTCCTCTCCTTCCTTCTC  GTCCTCCTCTCCTTCCTTCTCATGAGGAGGACAGCTGA  AGCGTGTAAACATCCTACACT  TCCTCCTCTCCTTCCTTCTC  GTCCTCCTCTCCTTCCTTCTCATGAAGGACAGCTGA  CTCGCTTCGGCAGCACA |
| U6 Reverse  U6 RT | AACGCTTCACGAATTTGCGT  AACGCTTCACGAATTTGCGT |
| Cel-miR-39-3p  Forward | GGGTCACCGGGTGTAAATC |
| Cel-miR-39-3p  Reverse  Cel-miR-39-3p  RT  STAT1 Forward  STAT1 Reverse  BTG2 Forward  BTG2 Reverse  GAPDH Forward  GAPDH Reverse | GAGAGGAGAGGAAGAGGGAA  GAGAGGAGAGGAAGAGGGAA  TGCTCCCTCTCTGGAATG  CTCCTTGCTGATGAAGCC  GGGAAACACTGGGAAGAAG  TGGAGAGACATGGGGAAG  ACAACTTTGGTATCGTGGAAGG  GCCATCACGCCACAGTTTC |

**Table S2** The sequence of si-STAT1

| Name | Sequences (5'–3') |
| --- | --- |
| STAT1 siRNA | CTGGAAGATTTACAAGATGAA |
